# Design and Analysis of Impedance Pumps Utilizing Electromagnetic Actuation

**DOI:** 10.3390/s100404040

**Published:** 2010-04-21

**Authors:** Yu-Hisang Wang, Yao-Wen Tsai, Chien-Hsiung Tsai, Chia-Yen Lee, Lung-Ming Fu

**Affiliations:** 1 Department of Mechanical and Automation Engineering, Da-Yeh University, 515 Changhua, Taiwan; 2 Department of Vehicle Engineering, National Pingtung University of Science and Technology, 912 Pingtung, Taiwan; 3 Department of Materials Engineering, National Pingtung University of Science and Technology, 912 Pingtung, Taiwan

**Keywords:** electromagnetic actuator, impedance pump, magnetic PDMS diaphragm, MEMS

## Abstract

This study designs and analyzes an impedance pump utilizing an electromagnetic actuator. The pump is designed to have three major components, namely a lower glass substrate patterned with a copper micro-coil, a microchannel, and an upper glass cover plate attached a magnetic PDMS diaphragm. When a current is passed through the micro-coil, an electromagnetic force is established between the coil and the magnetic diaphragm. The resulting deflection of the PDMS diaphragm creates an acoustic impedance mismatch within the microchannel, which results in a net flow. In performing the analysis, simulated models of the magnetic field, the diaphragm displacement and the flow rate are developed using Ansoft/Maxwell3D, ANSYS FEA and FLUENT 6.3 CFD software, respectively. Overall, the simulated results reveal that a net flow rate of 52.8 μL/min can be obtained using a diaphragm displacement of 31.5 μm induced by a micro-coil input current of 0.5 A. The impedance pump proposed in this study provides a valuable contribution to the ongoing development of Lab-on-Chips (LoCs) systems.

## 1. Introduction

In recent decades, rapid advances in micro-electro-mechanical systems (MEMS) have enabled the development of a wide variety of microfluidic devices for chemical control, mixing and analysis. Typically, the devices are designed to perform specific function such as cell sorting and counting, sample injection, specific mixing and so forth. As MEMS techniques have matured, the applications have been combined and implemented on a single chip, resulting in the emergence of LoCs systems. In realizing such systems, micropumps play an essential role in transporting precise volumes of sample fluid through the various components of the micro chips.

Micropumps can broadly be classified as either static, piezoelectric, or electromagnetic, depending upon their mode of actuation [1–13]. Zhu *et al.* [1] utilized a sol-gel method to fabricate thin piezoelectric films for the actuation of micro-cantilever arrays in hard disk devices. Meanwhile, Xu et al. [2] proposed a piezoelectric actuator based on a monolithic Pb(ZrTi)O_3_ layer for high-precision positioning applications. Alternatively, electromagnetic actuators represent an ideal solution for many modern MEMS-based applications with their simple driving mode, low actuation frequencies, large displacements and planar structures. Liu *et al.* [4] developed an active MEMS-based fluid control system incorporating surface micromachined magnetic actuators, and showed that the actuators were capable of achieving a large deflection (100 μm) under the application of a magnetic force with a magnetic flux density of 1.76 kGauss at 2.5 A current input. Lagorce *et al.* [5] presented a micro actuator based on a polymer magnet, and demonstrated that a good agreement existed between its theoretical and experimental response. However, the use of the device was limited with its maximum deflection of just 20 μm as a pumping component for practical microfluidic systems. In 2005, Hickerson *et al.* [6] proposed a valveless impedance pump in which a net flow was induced by periodically pinching a flexible section asymmetrically from its ends. In their design, the optimized lengths of elastic and inelastic sections are 1.91 and 15.2 cm, respectively. Their experimental results showed the flow rates are sensitive to duty cycle and pinching frequency. In their study, the pump was also simulated and showed the wave speed traveling on the tube did not necessarily have the same velocity, nor must be in phase with the flow rate. The flow exiting the impedance pump is typically pulsatile and the net flow rate (−10.9∼9.0 mL/min) has a non-linear relationship to the frequency of activation with characteristic peaks and flow reversals. The same authors also constructed a one-dimensional wave model which predicted many of the characteristics exhibited by the experiments of impedance pumping [7]. Yeo *et al.* [8] presented an impedance pump utilizing a PZT cantilever beam with a high frequency actuation. Their microchannel had the dimensions of 15 × 3 × 0.4 mm^3^, and a flow rate of 36 μL/s. Recently, Chang *et al.* [9] designed and analyzed a valveless impedance pump in which the actuation mechanism comprised a permanent magnet mounted on a flexible PDMS diaphragm positioned above a copper plated micro-coil at a height of 630 μm, corresponding to the position of the maximum electromagnetic force on the magnet. The valveless impedance pumping effect (Liebau Phenomenon) was first reported by Gerhart Liebau in 1954 and numerically examined by Bozi and Propst [14]. Based on partially elastic rigid walls, the impedance pump was operated by the interaction between traveling waves emitted from the compression and reflected waves at the impedance-mismatched positions, it exhibits a non-linear response to the actuating compression frequency and flow reversal with actuating frequencies at certain ranges. In their study, the theoretical results showed that a diaphragm deflection of 15 μm could be obtained by passing a current of 0.6–0.7 A through the micro-coil in order to produce a compression force of 11 μN. The design of the micropump was easily fabricated and was readily integrated with existing microfluidic chips due to its planar structure.

In 2008, Lee *et al.* [10] experimentally realized the design presented in [9] which resulted in an ideal solution with relatively low values of the excitation frequency and voltage for microfluidic systems in which relatively high pumping rates (*i.e.*, 7 mL/min) were required. The same research group [11] also presented a micro electromagnetic actuator with the maximum diaphragm deflection of 150 μm at an applied current of 0.6 A through a micro coil with a line width of 100 μm. Recently, for enhancing the performance of the micro impedance pump, Chang *et al.* [12] designed, analyzed and optimized the micro impedance pump and found a target diaphragm deflection of 20 μm could be obtained using a compression force of 12 μN developed by a micro-coil input current of 0.8 A.

However, despite the detailed analyses and optimized results of electromagnetic actuators and experimental ones of impedance pumps presented in the previous studies, the problem of numerically analyzing impedance pumps for enhancing their performance has attracted relatively little attention in the literature. Accordingly, the present study designs and analyzes an impedance pump utilizing a micro electromagnetic actuator featuring a magnetic PDMS diaphragm and a glass substrate patterned with a copper micro coil. The electrical current through the micro coil induces a magnetic force between the coil and the magnet electroplated on the PDMS diaphragm which causes the diaphragm to deflect, thereby creating an actuation effect. The periodic volume caused by the actuation effect produces a large stroke volume resulting in a flow in the channel due to the impedance effect. A series of analyzed trials are performed to investigate the pumping performance with various geometry parameters. The relationships between the coil current and the membrane displacement are then systematically examined. Finally, the analyzed results confirm that a net flow rate of 52.8 μL/min can be obtained using a diaphragm displacement of 31.5 μm induced by a micro-coil input current of 0.5 A.

## 2. Designs

**Figure 1.**
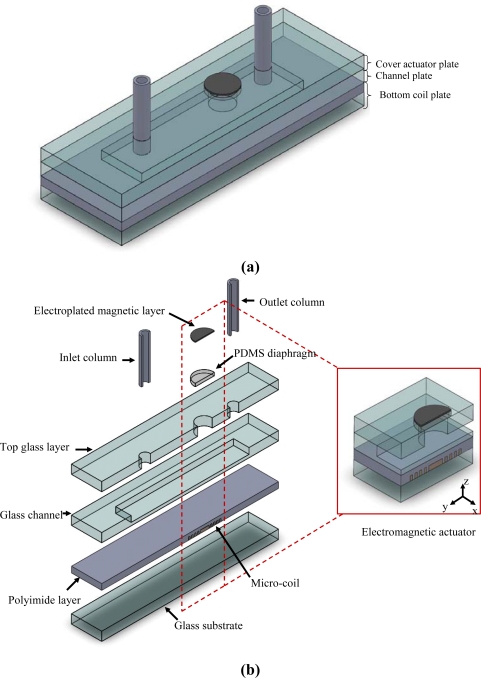
(a) Schematic illustration of valveless impedance pump. (b) Details of (a) [13].

As shown in Figure 1(a), the impedance pump comprises three basic plates, namely a bottom coil plate containing a planar micro-coil, a channel plate and a cover actuator plate with a PDMS diaphragm electroplated a magnetic layer on its upper surface. The pump body has overall dimensions of 26 mm × 15 mm × 2.5 mm (length × width × height), while the microchannel measures 11.35 mm × 4 mm × 50 μm (length × width × height). The present study designed three different micro-coils for evaluation purposes. In each case, the coil contained 10 turns and had a thickness and inner radius of 20 μm and 2,000 μm, respectively. Meanwhile, the widths, spacing, and outer radii of the three coils were specified as follows: (a) 125 μm / 150 μm / 9,500 μm; (b) 100 μm / 125 μm / 8,500 μm; and (c) 75 μm / 90 μm / 7,500 μm [13].

When a sinusoidal electrical current is passed through the micro-coil, a magnetic induction field is generated, which creates an electromagnetic force between the coil and the electroplated magnetic layer on the upper surface of the PDMS diaphragm. As a result, the diaphragm deflects bi-directionally, causing a periodic volume change of the channel with a frequency equivalent to that of the applied voltage. In the impedance pump, due to the interaction between traveling waves emitted from the compression and reflected waves at the impedance-mismatched positions, it not only has a non-linear response to the actuating compression frequency, but also shows reversal of flow direction under certain frequency ranges [14]. Ideally, the flow rate can be increased by increasing the stroke volume of the diaphragm. However, in designing the electromagnetic actuator, the volume change consistent with the required flow rate and its excitation frequency can not exceed the elastic limits of the PDMS diaphragm and the fundamental frequency of the actuator structure, respectively [9,13].

## 3. Analysis

### 3.1. Magnetic Analysis

In the electromagnetic actuation mechanism, the magnet is electroplated on the PDMS diaphragm which is positioned such that its center coincides with the vertical centerline of the micro coil. When an electrical current is passed through the coil, the resulting electromagnetic force acting on the diaphragm is given by equation (1) [15]:

(1) $$F_{z}=B_{r}\cdot \int_{z}^{z+h_{m}}S_{m}\cdot \frac{\partial H_{z}}{\partial z}\cdot \mathrm{dz}\approx B_{r}\cdot V_{m}\cdot \frac{\partial H_{z}}{\partial z},$$

where *H_z_* is the vertical magnetic field produced by the coil, *B_r_* is the retentivity of the magnet, and *S_m_*, *h_m_* and *V_m_* are the surface area, thickness and volume of the magnet, respectively. From Figure 1, it can be inferred that *H_z_* is symmetrical about both the x- and the y-axes. Equation (1) indicates that the magnitude of the magnetic force varies with the rate of change of the magnetic field in the vertical direction. To maximize the diaphragm deflection, the magnetic diaphragm should be positioned such that it coincides with the point in the magnetic field at which the gradient attains its maximum value. In the current study, this position is identified by evaluating the magnetic field, *H_z_*, and the gradient of the magnetic field, ∂*H_z_* / ∂*z*, numerically using the Ansoft/Maxwell 3D FEA software [10].

## 3.2. Actuator Displacement Analysis

**Figure 2.**
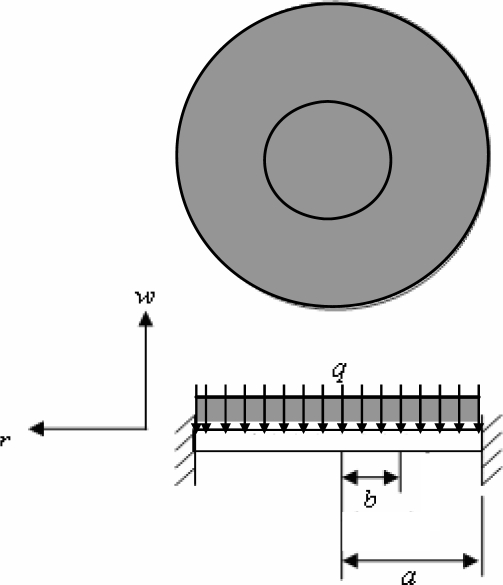
Fixed-edge circular diaphragm with a uniform load distributed over a central circular area with radius 0 < *r* < *a* [9,10,12,17].

In the current study, PDMS was specifically chosen as the diaphragm material since it has good flexibility characteristics, excellent biological compatibility and a high yield strength (elastic modulus *E* = 750 kPa, Poisson’s ratio *ν* = 0.5 and yield strength σ*_y_* = 20 kPa) [16]. Therefore, the diaphragm ensures a safe yet efficient pumping operation even under resonance conditions. In Figure 1, the maximum deflection takes place in the center of the diaphragm. Since the load imposed by the magnet is uniformly distributed, the diaphragm experiences a deflection over the circular area corresponding to the position of the magnet (Figure 2). The analysis commenced by considering the general case in which the load is uniformly distributed over a circle of radius *b* (0 < *b* < *a*). From Figure 2, the displacement field of the diaphragm is given respectively by (2) [9, 10, 12, 17]:

(2) $$w_{1}^{\prime }=\frac{P}{8\pi D}\cdot \left(-\left(r^{2}+b^{2}\right)\cdot \text{ln}\frac{a}{b}+\left(r^{2}-b^{2}\right)+\frac{1}{2}\left(1+\frac{b^{2}}{a^{2}}\right)\cdot \left(a^{2}-r^{2}\right)\right),$$

where *P* is the total load applied over the circle; *a* is the radius of the diaphragm; and *D* = *Eh*^3^ / 12(1 − *ν*^2^) is the flexural rigidity of the diaphragm, in which *E*, *ν* and *h* are the elastic modulus, Poisson’s ratio and thickness of the diaphragm, respectively.

The diaphragm displacement field induced by a uniform load *q* acting over the central circular area of radius *a* can be obtained by substituting *P* = 2*πbqdb* into Equation (2) and then integrating from 0 to *a* with respect to *b*. The displacement field of the diaphragm is therefore given by (3) [9,10,12,17]:

(3) $$w_{1}=\frac{{\mathrm{qa}}^{2}}{8D}\cdot \left(-\frac{r^{2}}{4}-\frac{a^{2}}{8}\right).$$

The maximum deflection of the loaded region of the diaphragm at radius 0 can be obtained by setting the radius parameter *r* equal to 0 in Equation (3), *i.e*.:

(4) $$w_{0}=\frac{-{\mathrm{Fa}}^{2}}{16\pi D},$$

where *F* is the electromagnetic force generated between the electroplated magnet and the micro coil and can be calculated either from Equation (1) or by using the Ansoft/Maxwell3D software.

### 3.3. Pumping Analysis

The physical model of the impedance pump developed in the present study utilizes a two-dimensional model [18] with the Navier-Stokes equations, which are written as:

(5) $$\frac{\partial v}{\partial t}+\nabla \cdot (\text{vv})=-\frac{1}{\rho }\nabla p+v\nabla^{2}v+\rho g$$

(6) ∇·v=0

In the equations above, v is the flow velocity in *x-y* plane, *p* is the pressure. In the simulation, the actuation displacement is given as by (7) [17]:

(7) $$\begin{array}{lll} \zeta (x,y,t) & =w_{0}\;\text{sin}(2\pi f){\left(1-{\left(\frac{\sqrt{x^{2}+y^{2}}}{R}\right)}^{2}\right)}^{2} & \text{within the magnetic film}\\ & =0 & \text{outside the film} \end{array}$$

**Figure 3.**
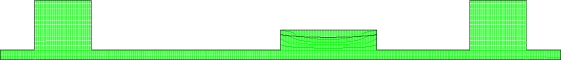
Meshes used for CFD simulation.

The actuation amplitude, *w_0_*, was calculated in equation (4) at various applied electrical current. Non-uniform meshes are generated over the pumping chamber, the buffers and channels for the simulation, which are illustrated in Figure 3. In order to capture the detailed flow fields, the meshes are refined in the junctions between the chamber and the channels, and the junctions between the buffers and the channel ends. A mesh independent analysis was conducted to ensure reasonable results. The simulations were carried out by using the FLUENT 6.3 CFD software. The pressure at the pump inlet and outlet were set to be equal, and the pumping flow rates were calculated by averaging the periodic flow rates over one period.

## 4. Results and Discussion

The magnet considered in the study is a CoNiMnP electroplated magnet [13] with a magnetic coercivity of 47.7 kA/m and a magnetic remanence of 0.2 Tesla [19]. When a current is applied to the micro-coil located beneath the central axis of the magnetic diaphragm, the direction of the magnetic field generated by the coil coincides with the direction of the magnet alignment and an attractive force can be produced. On the contrary, a repelling force is produced as the direction of the magnetic field of the coil is reversed.

In the current study, the radius of the PDMS diaphragm is 4,000 μm and its thickness is designed to be 30, 80 and 200 μm, respectively. Meanwhile, the electroplated magnet should be designed to possess the largest remanence and appropriate dimensions to obtain a sufficient magnetic force and to avoid influencing the safe operation of the diaphragm with a low stiffness. Therefore, the magnet thickness is specified to be 60, 110 and 170 μm, respectively.

**Figure 4.**
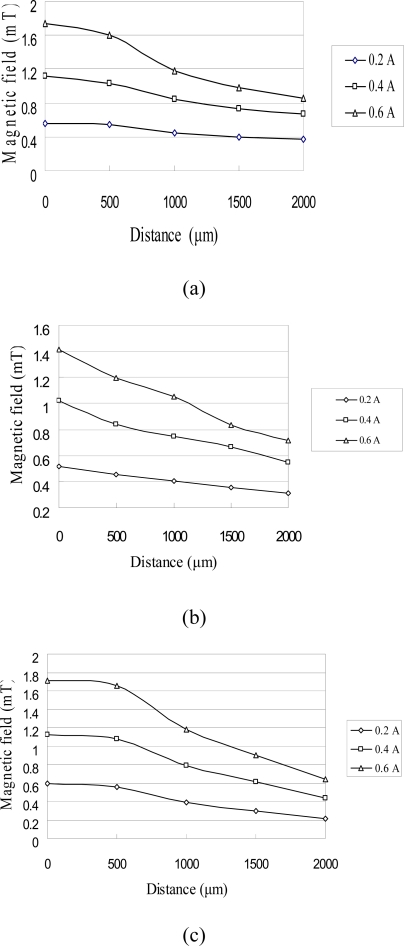
Variation of flux density with distance as function of input current for micro-coils with different widths: (a) 125 μm, (b) 100 μm and (c) 75 μm.

**Figure 5.**
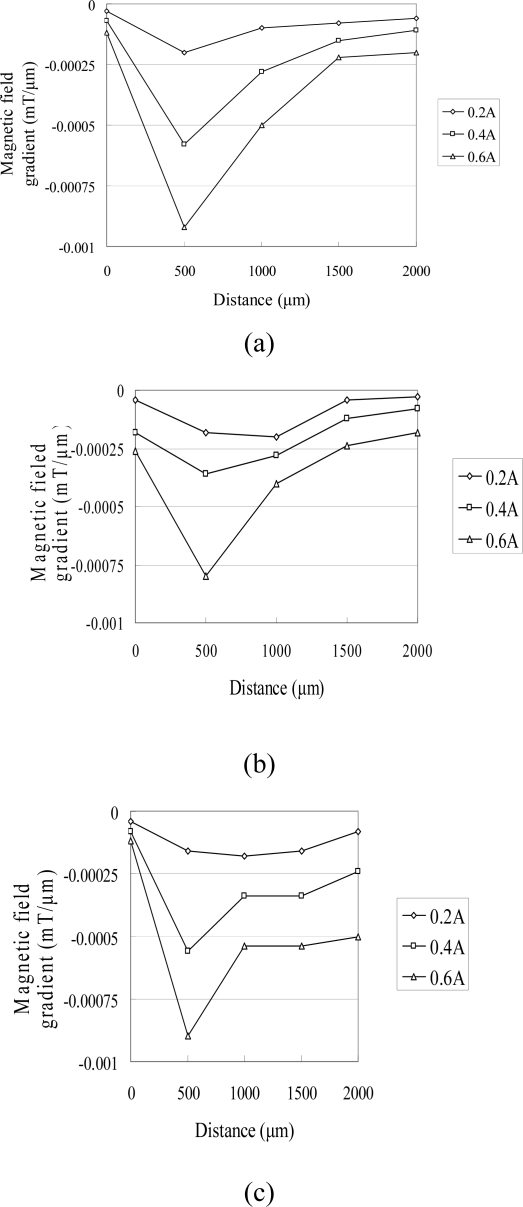
Variations of magnetic field gradient with distance as function of input current for micro-coils with different widths: (a) 125 μm, (b) 100 μm and (c) 75 μm.

In the characterization tests, the coils were supplied with input currents of 0.2, 0.4, and 0.6 A and the variation of the resulting flux density was simulated in the vertical direction along the central axis of the coil using the Ansoft/Maxwell3D FEA software. Figure 4 shows the simulation results obtained for the three micro-coil designs. It can be seen that the intensity of the magnetic field increases as the input current increases in every case. Furthermore, it can be observed that the intensity of the magnetic flux increases as the coil width decreases due to the corresponding enhancement of the concentration of the magnetic flux. Figure 5 illustrates the variation in the rate of change in the magnetic flux density along the vertical centerline of the micro-coils as a function of the input current. It can be found the performance of the electromagnetic actuator can be enhanced as the magnetic PDMS diaphragm is positioned at a height corresponding to the maximum rate of change of the magnetic flux. From an inspection of Figure 5, it is therefore concluded that the magnetic diaphragm should be positioned at a height of 500 μm above the planar surface of the micro-coils [13].

**Figure 6.**
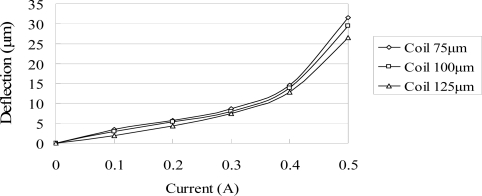
Variation of maximum diaphragm deflection with input current as function of coil width without driving liquid.

**Figure 7.**
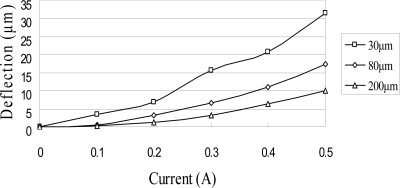
Variation of maximum diaphragm deflection with input current as function of PDMS diaphragm thickness without driving liquid.

**Figure 8.**
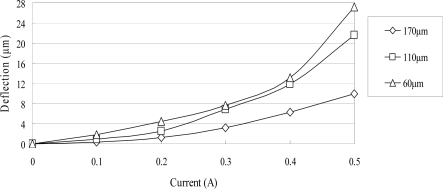
Variation of maximum diaphragm deflection with input current as function of magnetic layer thickness without driving liquid.

The ANSYS FEA software was used to model the deflection at the center of the magnetic diaphragm and it was found the deflection ranged from 1.8 to 31.5 μm in the current range of 0.2–0.5 A. Figure 6 presents the simulation results for the three micro-coil fabricated in the current study. A maximum displacement of 31.5 μm is obtained using a coil current of 0.5 A. It is also observed that for a constant coil current, the displacement increases as the coil width decreases due to the greater concentration of the magnetic flux. Figure 7 shows the variation of the maximum diaphragm displacement with the input current as a function of the PDMS diaphragm thickness. It can be seen that for a constant coil current, the diaphragm displacement increases with a reducing PDMS diaphragm thickness due to the corresponding reduction in the stiffness of the diaphragm. Figure 8 shows the correlation between the maximum diaphragm displacement and the magnetic layer thickness. For a constant coil current, it is observed that the diaphragm displacement increases with a reducing magnetic layer thickness.

Overall, Figures 7 and 8 confirm that the thickness dimensions of the PDMS diaphragm and the electroplated magnetic layer have a critical effect on the diaphragm displacement and therefore play a crucial role in determining the overall performance of the impedance pump. Though the magnetic force is proportional to the magnetic layer thickness [Equation (1)], the diaphragm displacement is inversely proportional to the cube of the magnetic layer thickness [12]. It is apparent that the thinner the magnetic layer is, the bigger the diaphragm displacement is. In the study, the optimal thickness of the magnetic layer is 30 μm because the magnetic property is poor as the thickness of the electroplated magnetic layer is less than 30 μm.

**Figure 9.**
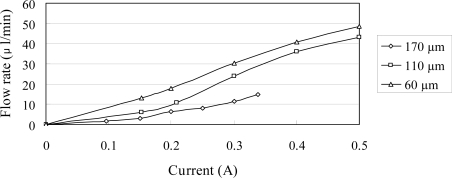
Variation of flow rate with input current as function of magnetic layer thickness at constant actuation frequency of 240 Hz.

Figure 9 shows the relationship between the flow rate and the input current for a constant excitation frequency of 240 Hz and various magnetic layer thicknesses. The results show that the flow rate increases approximately linearly as the input current increase. Furthermore, the flow rate increases with a decreasing magnetic layer thickness due to the corresponding improvement in the change of traveling wave amplitude induced within the microchannel of the impedance pump.

**Figure 10.**
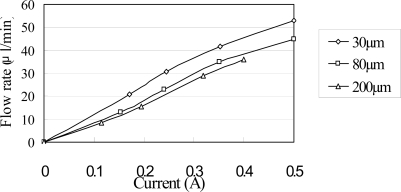
Variation of maximum diaphragm deflection with input current as function of PDMS diaphragm thickness at constant actuation frequency of 240 Hz.

Figure 10 illustrates the variation in the flow rate with the input power for a constant magnetic layer thickness of 60 μm and three different PDMS diaphragm thicknesses in the range of 30–200 μm. In accordance with general thin plate theory, the diaphragm stiffness reduces with a reducing diaphragm thickness. Thus, as shown in Figure 10, the flow rate increases as the PDMS diaphragm thickness reduces. From inspection, a maximum flow rate of 52.8 μL/min is obtained at an input current of 0.5 A and a PDMS diaphragm thickness of 30 μm. In Ref [13], the experimental result of corresponding flow rate is 1.5 μL/s at the same input current and PDMS diaphragm thickness. Both the simulated and experimental results show high correspondence for periodic flow conditions.

## 5. Conclusions

This study had designed and analyzed an impedance pump incorporating an electromagnetic actuator comprising a magnetic PDMS diaphragm and a planar micro-coil. The theoretical design models have been numerically validated using the Ansoft/Maxwell3D, ANSYS and FLUENT 6.3 CFD simulation softwares. The numerical results have shown that the actuator provides a large diaphragm deflection, allow the flow rate to be flexibly controlled, and can be excited using a low electrical current and a low frequency. In addition, it has been shown that the maximum flow rate is 52.8 μL/min and is obtained using an actuating current of 0.5 A and a frequency of 240 Hz. The micropump can easily be fabricated using MEMS techniques and has a planar structure which therefore can be readily integrated with other microfluidic devices to realize LoCs systems.
